# Systematic review of models assessing the economic value of routine varicella and herpes zoster vaccination in high-income countries

**DOI:** 10.1186/s12889-015-1861-8

**Published:** 2015-06-05

**Authors:** Oliver Damm, Bernhard Ultsch, Johannes Horn, Rafael T. Mikolajczyk, Wolfgang Greiner, Ole Wichmann

**Affiliations:** Department of Health Economics and Health Care Management, Bielefeld School of Public Health, Bielefeld University, Bielefeld, Germany; Immunisation Unit, Robert Koch Institute, Berlin, Germany; Helmholtz Centre for Infection Research, Brunswick, Germany; Hannover Medical School, Hannover, Germany

**Keywords:** Cost-effectiveness, Economic evaluation, Vaccination, Varicella, Zoster, Systematic review

## Abstract

**Background:**

A systematic review was conducted to assess the cost-effectiveness of routine varicella and herpes zoster (HZ) vaccination in high-income countries estimated by modelling studies.

**Methods:**

A PubMed search was performed to identify relevant studies published before October 2013. Studies were included in the review if they (i) evaluated the cost-effectiveness of routine childhood or adolescent varicella vaccination and/or HZ vaccination targeting the elderly, and if they (ii) reported results for high-income countries.

**Results:**

A total of 38 model-based studies were identified that fulfilled the inclusion criteria. Routine childhood or adolescent varicella vaccination was cost-effective or cost-saving from a payer perspective and always cost-saving from a societal perspective when ignoring its potential impact on HZ incidence due to reduced or absent exogenous boosting. The inclusion of the potential impact of childhood varicella vaccination on HZ led to net quality-adjusted life-year (QALY) losses or incremental cost-effectiveness ratios exceeding commonly accepted thresholds. Additional HZ vaccination could partially mitigate this effect. Studies focusing only on the evaluation of HZ vaccination reported a wide range of results depending on the selected target age-group and the vaccine price, but most found HZ vaccination to be a cost-effective or marginally cost-effective intervention. Cost-effectiveness of HZ vaccination was strongly dependent on the age at vaccination, the price of the vaccine, the assumed duration of protection and the applied cost per QALY threshold.

**Conclusions:**

While HZ vaccination is mostly considered cost-effective, cost-effectiveness of varicella vaccination primarily depends on the in- or exclusion of exogenous boosting in the model. As a consequence, clarification on the role of exogenous boosting is crucial for decision-making regarding varicella vaccination.

## Background

Primary infection with varicella-zoster virus (VZV) causes varicella (chickenpox), which occurs mainly in childhood [1, 2]. The virus persists lifelong in the dorsal roots of the spinal and cranial ganglia. Later in life the virus can be reactivated, manifesting as shingles (herpes zoster, HZ), a painful skin rash that lasts approximately one month [1, 3]. The main complication of HZ is postherpetic neuralgia (PHN), a long lasting neuropathic pain in the area formerly affected by the HZ rash [3–6].

Live-attenuated monovalent varicella vaccines or combination vaccines against measles, mumps, rubella and varicella (MMRV) licensed for use in children are available in most industrialised countries. As of today there is one HZ vaccine licensed for individuals aged 50 years and older.

Routine childhood varicella vaccination is generally recommended in the United States, Australia, Canada, Qatar, Saudi Arabia, Republic of Korea, Taiwan, Uruguay and several countries in Europe including Germany, Greece, Finland and parts of Italy and Spain [7, 8]. A significant decline in varicella incidence was observed after the introduction of routine vaccination in several countries [8–11]. Nationwide vaccination recommendations for the prevention of HZ currently exist in Austria for individuals aged 50+ [12], the United States and in Canada for individuals aged 60+ [13, 14] and in the UK for individuals aged 70–79 [15].

In the 1960s, Hope-Simpson hypothesised that after primary infection a re-exposure to wild-type VZV would sub-clinically boost the individual’s VZV-specific immunity, thereby suppressing VZV reactivation and decreasing the probability of developing HZ [16]. Since then, the hypothesis that HZ incidence will increase in a population with routine varicella vaccination has been discussed in the literature [17]. According to models, varicella vaccination might lead to a substantial increase in HZ incidence during approximately 40–50 years after initiation of routine vaccination [18, 19]. In the United States, where routine varicella vaccination was introduced in 1995, studies monitoring HZ incidence have reported inconsistent results until today [20]. However, a more recent systematic review based on 39 multidisciplinary studies concluded that exogenous boosting exists, but its extent and public health impact remain unclear [21].

Our objective was to assess the cost-effectiveness of routine varicella and HZ vaccination in high-income countries estimated by modelling studies.

## Methods

### Search strategy

A PubMed search was performed to identify English- and German-language articles on economic evaluations of varicella and HZ vaccination published before October 2013. The systematic literature search was conducted using the following key words: (varicella OR chickenpox OR herpes zoster OR shingles OR varicella-zoster OR VZV OR “Chickenpox” [MeSH] OR “Herpes Zoster” [MeSH]) AND (vaccination OR vaccine OR vaccinating OR vaccinate OR vaccinated OR immunisation OR immunization OR “Vaccination” [MeSH] OR “Vaccine” [MeSH] OR “Chickenpox Vaccine” [MeSH] OR “Herpes Zoster Vaccine” [MeSH]) AND (economic OR economics OR cost OR costs OR cost-effectiveness OR cost-effective OR cost-utility OR cost-benefit OR benefit-cost OR cost-saving OR pharmacoeconomic OR pharmacoeconomics OR ICER OR QALY OR “Costs and Cost Analysis” [MeSH]). Application of the non-MeSH search terms was restricted to titles and abstracts of the PubMed records. In addition, we screened reference lists of all included studies to identify further articles of interest.

### Study selection

Titles and abstracts of the obtained search results were screened independently by two reviewers. Full-text versions of all potentially relevant studies were retrieved and assessed according to pre-defined inclusion and exclusion criteria by the same two reviewers. Any disagreements between reviewers on inclusion of particular studies were resolved by consensus. A study was included if it was a full or partial economic evaluation of routine childhood (or adolescent) varicella vaccination or a HZ immunisation scheme targeting the elderly and if the modelling study reported results for a high-income country as specified by the World Bank (high-income OECD-members) [22]. Inclusion criteria related to comparators included no vaccination as well as existing vaccination programmes or private coverage. We did not define inclusion or exclusion criteria related to outcome measures. We excluded non-original research papers (i.e. review articles, letters, and editorials), studies that focused on vaccination of specific target groups (e.g. health care workers, adults without history of chickenpox, transplant patients, seronegative postpartum women, army recruits and cadets, immigrants and refugees), studies that did not provide sufficient details on the applied methods, studies that evaluated only combined strategies of serotesting and vaccination as well as studies that evaluated combination vaccines without reporting separate results for each component.

### Data extraction and synthesis

Critical appraisal of all included studies was undertaken by using the framework for quality assessment of decision-analytic models proposed by Philips et al. [23]. The quality assessment was performed by two independent reviewers. The framework used considers aspects related to structure, data, and consistency of health economic models. The following information was systematically extracted from each included study: citation details, country, characteristics of the vaccination programme (e.g. target age group, vaccine type, vaccine efficacy, vaccination coverage), main features of the modelling approach (e.g. model type, time horizon, interaction between varicella and HZ), characteristics of the economic analysis (e.g. determination of the perspective, choice of discount rate, valuation of health gains), key findings as well as funding sources. The economic value of routine varicella and HZ vaccination was assessed by comparing incremental cost-effectiveness ratios (ICERs) and/or benefit-cost ratios (BCRs) among studies taking into account different immunisation strategies, perspectives as well as clinical and epidemiological features of VZV.

To improve comparability between studies and across countries, all cost estimates were inflated to 2010 values (latest price year used in included studies) applying country-specific consumer price indices and converted to Euros with the German level of purchasing power using purchasing power parities obtained from the Organisation for Economic Co-operation and Development (OECD) [24].

The reporting of this systematic review was performed in accordance with the PRISMA statement [25]. However, not all items of the PRISMA statement checklist are applicable to economic evaluations.

## Results

### Search results and quality assessment

**Fig. 1:**
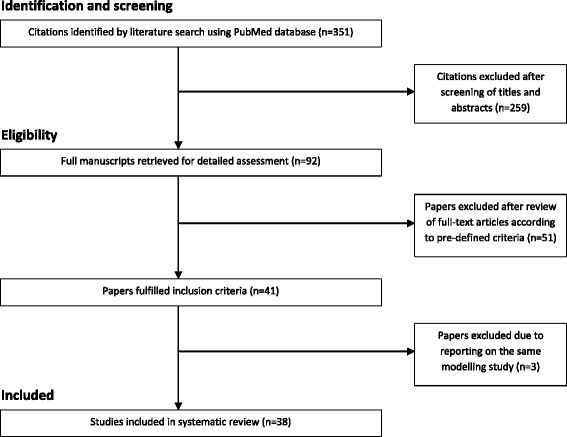
Flow chart of study selection process

The literature search in PubMed identified 351 articles. After screening the titles and abstracts of these hits, 92 papers were considered for full-text review. 41 papers of the obtained full-text articles met the inclusion criteria. The main reasons for exclusion were incorrect type of study (such as review articles), incorrect intervention or studies assessing the vaccination of specific target groups. Few studies were excluded due to insufficient information on the methods used. Four of the 41 papers that met the inclusion criteria reported on the same modelling study and three of them were therefore excluded. Finally, 38 studies were included in the review. A flow chart of the study selection process and the corresponding results is outlined in Fig. 1.

Most of the included studies were of high quality. However, some studies evaluating varicella vaccination did not use a dynamic modelling approach and hence are not able to capture indirect effects of vaccination. Most studies performed probabilistic sensitivity analysis to address the issue of parameter uncertainty, but only a few studies evaluated the impact of structural uncertainties. Several studies also lack detailed descriptions/discussion of the sources of utility weights.

### Study characteristics

#### Varicella vaccination

We included 23 model-based studies evaluating the cost-effectiveness of routine varicella vaccination. Table 1 gives an overview of general study characteristics and information on the applied modelling framework of these studies. The majority of the studies was performed for European countries.

**Table 1:** General study characteristics of the models evaluating routine varicella vaccination

Study	Country	Model type	Accounting for herd protection	Impact of varicella vaccination on HZ incidence	Time horizon	Type of economic evaluation	Perspective	Discount rate (costs/health effects)	Costing year and currency	Funding source
Banz et al. [35]	Germany	Dynamic	Yes	No	30 years	CC	Health care payer; societal	5 %/0 %	1999 EUR	Industry
Banz et al. [51]	Switzerland	Dynamic	Yes	No	30 years	CC; CEA	Health care payer; societal	5 %/0 %	2008 CHF	Industry
Beutels et al. [37]	Germany	Static	No	No	70 years	CC; CEA	Health care payer; societal	5 %/5 %	1995 DEM	Independent
Bilcke et al. [30]	Belgium	Dynamic	Yes	Depending on the type of analysis	Various	CEA; CUA	Health care payer	3 %/1.5 %	2010^a^ EUR	Independent
Bonanni et al. [36]	Italy	Dynamic	Yes	No	30 years	CC	Health care payer; societal	3 %/0 %	2007^a^ EUR	Industry
Brisson et al. [31]	Canada	Dynamic	Yes	Yes (only in a separate analysis)	30 years	CC; CEA	Health care payer; societal	3 %/3 %	1997/1998 CAD	Independent
Brisson et al. [32]	England and Wales	Dynamic	Yes	Yes	80 years	CUA	Health care payer; societal	3 %/3 %	2001 GBP	Independent
Coudeville et al. [52]	France	Dynamic	Yes	No	30 years	CC	Health care payer; societal^b^	5 %/NA	1995 FRF	Industry
Coudeville et al. [53]	Italy	Dynamic	Yes	No	50 years	CC	Health care payer; societal	3 %/NA	2002 EUR	Industry
Coudeville et al. [54]	France and Germany	Dynamic	Yes	No	50 years	CC; CEA	Health care payer; societal	3 %/3 %	2002 EUR	Industry
Diez Domingo et al. [55]	Spain	Static	No	No	20 years	CC	Health care payer; societal	5 %/NA	1994 PTA	Independent
Getsios et al. [56]	Canada	Static	No	No	70 years	CEA; CUA	Health care payer; societal	3 %/3 %	1998 CAD	Independent
Ginsberg & Somekh [57]	Israel	Static	Partially	No	Lifetime	CC	Health care payer; societal	3 %/NA	2002 USD	Independent
Hammerschmidt et al. [58]	Germany	Dynamic	Yes	No	30 years	CC	Health care payer; societal	Not specified (probably 5 % for costs)	1999/2006^c^ EUR	Industry
Huse et al. [59]	USA	Static	No	No	24 years	CC	Societal	5 %/5 %	1991 USD	Industry
Lenne et al. [60]	Spain	Dynamic	Yes	No	50 years	CC; CEA	Health care payer; societal	3 %/0 %	2004 EUR	Industry
Lieu et al. [61]	USA	Dynamic	Yes	No	30 years	CC; CEA	Health care payer; societal	5 %/5 %	1990 USD	Independent
Preblud et al. [62]	USA	Static	No	No	30 years	CC	Health care payer; societal	5 %/0 %	1984 USD	Independent
Scuffham et al. [63]	New Zealand	Static	No	No	30 years	CC	Health care payer; societal	5 %/5 %	1997 NZD	Industry
Scuffham et al. [34]	Australia	Static	No	No	30 years	CEA	Health care payer	5 %/5 %	1996/1997 AUD	Independent
Thiry et al. [64]	Italy	Static	No	No	100 years	CC; CEA	Health care payer; societal	3 %/3 %	2002 EUR	Industry
van Hoek et al. [33]	UK	Dynamic	Yes	Yes	Infinite	CUA	Health care payer	3.5 %/3.5 %	2007 GBP	Independent
Zhou et al. [29]	USA	Static	No	No	Lifetime	CC; CUA	Health care payer; societal	3 %/3 %	2006 USD	Industry

*CC* cost-comparison; *CEA* cost-effectiveness analysis; *CUA* cost-utility analysis; *NA* = not applicable

^a^Assumption

^b^Indirect costs were not evaluated in monetary terms but reported as the number of days of absence from work

^c^Personal communication with the author (1999 prices; 2006 vaccine prices)

13 studies used a fully dynamic modelling approach in terms of simulating the transmission dynamics of varicella. 10 studies were based on static models. By definition, all dynamic models accounted for herd protection effects. The static model from Israel reported to have included partial herd protection effects by use of an adjustment factor. Apart from herd protection effects, varicella immunisation programmes can induce other population-level effects such as the hypothesised increase in HZ incidence due to a reduced or absent exogenous boosting after varicella vaccine introduction and subsequent decrease in wild virus circulation in the population. Most of the models ignored the close relationship between varicella and HZ. Only four studies modelled the possible impact of routine varicella vaccination on HZ incidence in a population due to a decrease in wild-type VZV circulation.

Many studies performed a cost comparison analysis and reported the results as BCRs. Some of these studies claimed to have conducted a cost-benefit analysis but this would require a monetary valuation of health effects. However, the included studies which we classified as cost comparison analyses only considered costs and cost offsets instead of valuing health effects in monetary terms.

Most of the studies adopted both a health care payer and a societal perspective. Costs were discounted with a discount rate ranging from 3 to 5 %. The discount rate for health effects ranged from 0 to 5 %. Vaccination costs varied widely across studies. The simulated time horizons ranged from 20 years to a lifetime or an infinite time horizon, but most studies used a period of 30 or 50 years.

Aspects related to vaccine characteristics and immunisation strategies considered in the included studies are presented in Table 2. The majority of the studies compared universal vaccination to a situation without vaccination. Few studies chose an existing one-dose varicella vaccination programme or a situation with low private vaccination coverage as comparator. While most of the studies considered a 1-dose vaccination schedule, some studies also (or exclusively) assessed the impact of two varicella vaccine doses. Considered vaccine efficacy ranged from 80 to 97 % and from 93 to 96 % for the first and the second dose, respectively. Waning of vaccine-induced immunity was modelled in very different ways. Some models did not consider waning immunity at all; other models used yearly waning rates of 0.5 or 3.1 %. A UK study used various waning rates ranging from 0.05 to 6.7 %. Some studies stated that waning was applied to 15 % of the protected vaccinees without quantifying the waning rate per year. Assumed vaccination coverage in the models ranged from 30 to 97.15 %. Most of the studies included a fee for administering the vaccine. Some studies included additional costs for treating adverse events and/or costs of vaccine wastage.

**Table 2:** Vaccine characteristics and immunisation strategies considered in the models evaluating routine varicella vaccination

Study	Age at vaccination	Vaccine efficacy	Waning (per year)	Vaccination coverage	Vaccination costs per dose (2010 EUR; German price level)
Banz et al. [35]	15 months; 11–12 years	86 %	0.5 %	85 % (children); 30 % (adolescents)	EUR 65.93 (children)^a^; EUR 71.38 (adolescents)^a^
Banz et al. [51]	1-2 years; 11–15 years	95 %	0.5 %	70 % (children); 85 % (adolescents)	EUR 42.20 (children)^a^; EUR 56.65 (adolescents)^a^
Beutels et al. [37]	15 months; 12 years	90 %	Waning in 15 % of the protected vaccinees^b^	70 %	EUR 50.82^a^
Bilcke et al. [30]	1 year (1st dose); 4, 6 or 11 years (2nd dose)	Data from van Hoek et al. 2012	Data from van Hoek et al. 2012	50 % or 95 % (1st dose); 50 %, 80 % or 90 % (2nd dose)	EUR 44.92^a^
Bonanni et al. [36]	12-18 months; 13 years	90 % (1st dose); 93 % (2nd dose)	3 %	85 %	EUR 46.81^a,c^
Brisson et al. [31]	12 months; 12 years	93 %	3.1 %	90 % (infants); 80 % (adolescents)	EUR 51.42 (children)^a^; EUR 68.57 (adolescents)^a^
Brisson et al. [32]	Infants; 11 years	93 %	3.1 %	90 % (infants); 80 % (adolescents)	EUR 44.32 (children)^a^; EUR 59.10 (adolescents)^a^
Coudeville et al. [52]	<6 years	90 %	Waning in 15 % of the protected vaccinees^b^	80 %	EUR 18.11^c,d^
Coudeville et al. [53]	12-36 months	97 %	3.1 %	45 %-90 %	EUR 52.00^a,c^
Coudeville et al. [54]	12-36 months	97 %	3.1 %	45 %-90 %	EUR 64.08 (Germany)^a,c^; EUR 59.56 (France)^a,c^
Diez Domingo et al. [55]	15 months	90 %	No waning	95 %	EUR 37.11
Getsios et al. [56]	12 months	90 %	Waning in 15 % of the protected vaccinees^b^	85 %	EUR 60.25^a,d^
Ginsberg & Somekh [57]	12 months	87.6 %	3.1 %	94 %	EUR 8.01^a,c,d^
Hammerschmidt et al. [58]	11-23 months (catch-up of 2–17 year olds)	86 % (1-dose schedule); 95 % (2-dose schedule)	0.5 %	90 % (1st dose); 80 % (2nd dose); 30 % (catch-up); 10 % (comparator)	EUR 47.38 (monovalent vaccine); EUR 47.92 (varicella-attributable cost of the MMRV vaccine)
Huse et al. [59]	15 months	95 %	No waning	Not specified	EUR 62.31^a^
Lenne et al. [60]	1-2 years	97 %	3,1 %	97,15 %	EUR 42.54^a^
Lieu et al. [61]	<6 years	90 %	Waning in 15 % of the protected vaccinees^b^	97 %	EUR 54.12^a^
Preblud et al. [62]	15 months	90 %	No waning	90 %	EUR 25.53
Scuffham et al. [63]	15 months	95 %	No waning	80 %; 10 % (comparator)	EUR 45.94
Scuffham et al. [34]	12 months; 12 years	95 %	No waning	80 % (infants); 50-75 % (adolescents)	EUR 42.26
Thiry et al. [64]	11 years	93,12 %	3.1 %	60 %	EUR 52.36^a^
van Hoek et al. [33]	1 year (1st dose); 3 years (2nd dose)	89-96 % (1st dose); 93-96 % (2nd dose)	1.5-6.7 % (1st dose); 0.05-2.6 % (2nd dose)	90 % (1st dose); 80 % (2nd dose)	EUR 41.19
Zhou et al. [29]	Children	80 % (1-dose schedule)^e^; 93 % (2-dose schedule)	No waning	Age-specific coverage rates; 95 % (2nd dose)	EUR 49.90 (monovalent vaccine)^f^; EUR 65.64 (MMRV vaccine)^f^

*MMRV* measles, mumps, rubella and varicella

^a^Including administration costs

^b^Waning rate per year not quantified

^c^Including costs of treating adverse events

^d^Including vaccine wastage

^e^This efficacy estimate was not directly used in the model; the vaccine-induced reduction in incidence was calculated by using age-specific surveillance data

^f^Public sector price

#### Herpes zoster vaccination

We included 17 model-based studies evaluating the cost-effectiveness of HZ vaccination. The main study characteristics are summarised in Table 3. We identified 15 studies which considered the health economic impact of HZ vaccination exclusively. The remaining two studies considered both varicella and HZ vaccination. Most studies were conducted for European countries. The two studies targeting varicella as well as HZ vaccination applied a dynamic modelling approach. One study used a discrete-event simulation model that simulated individual patients [26]. All other cost-effectiveness studies were based on static (Markov) state-transition models or similar models using single or multiple cohorts. However, the number of included disease states differed among models. While most models considered common states like healthy, HZ, PHN, and death, four studies included also different pain levels (mild, moderate and severe) for HZ and PHN. Some studies failed to report explicit information on the modelled health states or their number.

**Table 3:** General study characteristics of the models evaluating routine HZ vaccination

Study	Country	Model type	Time horizon	Type of economic evaluation	Perspective	Discount rate (costs/health effects)	Costing year and currency	Funding source
Annemans et al. [65]	Belgium	Static	Lifetime	CEA; CUA	Health care payer (with and without co-payments); societal	3 %/1.5 %	2007 EUR	Industry
Bilcke et al. [38]	Belgium	Static	Lifetime	CEA; CUA	Health care payer	3 %/1.5 %	2009^a^ EUR	Independent
Bilcke et al. [30]	Belgium	Dynamic^b^	Various	CEA; CUA	Health care payer	3 %/1.5 %	2011^a^ EUR	Independent
Bresse et al. [66]	France	Static	Lifetime	CEA; CUA	Health care payer (with and without co-payments)	4 %/4 %^c^	2010 EUR	Industry
Brisson et al. [67]	Canada	Static	Lifetime	CUA	Health care payer	5 %/5 %	2005 CAD	Industry
de Boer et al. [40]	Netherlands	Static	Up to 41 years	CUA	Health care payer; societal	4 %/1.5 %	2010 EUR	Independent
Edmunds et al. [68]	England and Wales	Static	Lifetime	CEA; CUA	Health care payer	3 %/3 %	1998 GBP	Independent
Hornberger et al. [28]	USA	Static	30 years	CUA	Societal	3 %/3 %	2006 USD	Independent
Moore et al. [69]	UK	Static	Lifetime	CEA; CUA	Health care payer; societal	3.5 %/3.5 %	2006 GBP	Industry
Najafzadeh et al. [26]	Canada	Static	Lifetime	CUA	Health care payer	5 %/5 %	2008 CAD	Independent
Pellissier et al. [70]	USA	Static	Lifetime	CUA	Health care payer; societal	3 %/3 %	2006 USD	Industry
Rothberg et al. [42]	USA	Static	Not specified	CUA	Societal	3 %/3 %	2005 USD	Independent
Szucs et al. [71]	Switzerland	Static	Lifetime	CEA; CUA	Health care payer; societal	3.5 %/1.5 %	2010^a^ CHF	Industry
Ultsch et al. [39]	Germany	Static	Lifetime	CEA; CUA	Health care payer; societal	3 %/3 %	2010 EUR	Independent
van Hoek et al. [72]	England and Wales	Static	Lifetime	CUA	Health care payer	3.5 %/3.5 %	2006 GBP	Independent
van Hoek et al. [33]	UK	Dynamic^b^	Infinite	CUA	Health care payer	3.5 %/3.5 %	2007 GBP	Independent
van Lier et al. [41]	Netherlands	Static	Not specified	CUA	Health care payer; societal	4 %/1.5 %	2008 EUR	Independent

*CEA* cost-effectiveness analysis; *CUA* cost-utility analysis

^a^Personal communication with the author or assumption

^b^Combined evaluation of varicella and HZ vaccination

^c^Discount rate for costs and health effects was reduced to 2 % after 30 years

All studies conducted a cost-utility analysis (CUA). Furthermore, almost half of the studies also performed a cost-effectiveness analysis (CEA). According to respective national guidelines, six studies used different discount rates for costs and health effects. One study used equal discount rates for costs and health effects that changed over time following the current French guidelines: A 4 % discount rate was used for the first 30 years of the model run and afterwards the discount rate was reduced to 2 %. Most studies used a lifetime horizon.

Details of the HZ vaccination-related input data are shown in Table 4. All models compared a vaccination scenario with no vaccination. Vaccine efficacy estimates were mostly based on clinical trial data of the Shingles Prevention Study [27]. Several studies neglected waning of vaccine-induced immunity in the base-case analysis. The age at vaccination varied between 50 and 80 years. Six studies did not report the assumed vaccination coverage. However, in static models vaccination costs and effects are proportional to vaccination coverage and hence the level of coverage has no impact on the ICER.

**Table 4:** Vaccine characteristics and immunisation strategies considered in the models evaluating routine HZ vaccination

Study	Age at vaccination (in years)	Vaccine efficacy against HZ	Waning or duration of protection	Vaccination coverage^a^	Vaccination costs per dose (2010 EUR, German price level)
Annemans et al. [65]	50+	37.6-63.9 %, age-dependent	No waning^b^	20 %	EUR 141.39^c^
Bilcke et al. [38]	60-85	Age-dependent (values are reported graphically only)	Consideration of waning depends on the choice of scenario	30 %	EUR 106.95^d^
Bilcke et al. [30]	50 or 60	77 %^e^	Duration of protection of 7.5 years or lifelong protection	30 % or 70 %	EUR 103.38^d^
Bresse et al. [66]	65+	18-64 %, age-dependent	4.15 % per year and vaccine efficacy was set to zero after 10 years	20 %	EUR 117^f^
Brisson et al. [67]	50-80	26-75 %, age-dependent	No waning^b^	Not specified	EUR 108.60
de Boer et al. [40]	60-75	41.2-69.4 %, age-dependent	8.3 % per year (= duration of protection of 12 years)	Not specified	EUR 89.10^d^
Edmunds et al. [68]	65	30-70 %	Duration of protection of 2.5 years to life long	60 %	EUR 122.13^d,g^
Hornberger et al. [28]	69	Modelled by applying age-specific incidence of HZ in vaccine and placebo-treated arm of the clinical study, age-dependent	Duration of protection of 30 years	Not specified	EUR 43.85-438.46^d,h^
Moore et al. [69]	50+	37.6-63.9 %, age-dependent	No waning^b^	40 %	EUR 143.28^d^
Najafzadeh et al. [26]	60+	Modelled by applying age-specific incidence of HZ in vaccine and placebo-treated arm of the clinical study, age-dependent	4.5 % per year	Not specified	EUR 101.83
Pellissier et al. [70]	60+	27.1-69.8 %, age-dependent	No waning^b^	Not specified	EUR 147.32^d^
Rothberg et al. [42]	60-80	Age-dependent	Waning considered but not quantified	Not specified	EUR 134.74^d^
Szucs et al. [71]	70-79	37.60-63.9 %, age-dependent	No waning^b^	20 %	EUR 143.09^d^
Ultsch et al. [39]	50-80	13.22-69.8 %, age-dependent	8.3 % per year following 10 years of stable vaccine efficacy	20 %	EUR 147.48^d^
van Hoek et al. [72]	60-75	31-95 % (based on 15 different take and waning scenarios), age-dependent	Duration of protection of 3.6-100 years (based on 15 different take and waning scenarios)	73.5 %	EUR 88.36^d^
van Hoek et al. [33]	75	Data from van Hoek et al. [72]	Data from van Hoek et al. [72]	70 %	EUR 86.37^d^
van Lier et al. [41]	60-80	Data from van Hoek et al. [72]	Duration of protection of 7.5 years	75 %	EUR 81.54^d^

*HZ* herpes zoster

^a^In static models vaccination costs and effects are proportional to vaccination coverage. Hence, the level of coverage has no impact on the incremental cost-effectiveness ratio

^b^Base-case analysis

^c^Including co-payments

^d^Including administration costs

^e^This value was assumed for the age group of 60–64 years. The supplemental material of this study also provides values for higher age groups (7-68 %) but no estimate is given for the age group below 60 years

^f^Reimbursement rate was assumed to be 65 % when taking a third-party payer perspective

^g^Costs of an immunisation course comprising two doses

^h^Including public awareness campaign, patient travel time, time receiving vaccine and costs of treating adverse events

A wide range of vaccination costs was applied across the studies. One study [28] considered vaccination costs between EUR 43.85 and 438.46 per dose in multiple scenarios, while the vaccination costs among the other studies ranged from EUR 81.54 to 147.48 per dose or per immunisation course. Thirteen studies included administration fees in these cost estimates. One study included additional costs for a public awareness campaign, patient time costs, and costs for treating adverse events.

### Results of the included studies

#### Varicella vaccination

The results of economic evaluations of varicella vaccination are summarised in Table 5. BCRs for **one-dose varicella vaccination of young children** ranged between 0.30 and 1.94 when taking a health care payer perspective and ignoring a potential impact on HZ. Six studies reported BCRs above 1 or stated that vaccination would lead to cost-savings. Eleven studies reported BCRs below 1 or calculated ICERs. In these studies, costs per life year gained (LYG) ranged between EUR 563 and EUR 40,193. One study reported a BCR of 1 which means that one-dose varicella vaccination was a cost-neutral intervention [29]. When adopting a societal perspective and ignoring a potential impact on HZ, all studies found that one-dose varicella vaccination of toddlers and young children would be cost-saving with BCRs ranging from 1.61 to 19.33.

**Table 5:** Economic results of the models evaluating routine varicella vaccination (2010 EUR, German price level)

Study	Age at vaccination	Dose schedule	Comparator	Health care payer perspective	Societal perspective
Banz et al. [35]	15 months	1	No vaccination	BCR 1.75	BCR 4.12
	11-12 years^a^	1	No vaccination	BCR 1.13	BCR 8.44
	combined	1	No vaccination	BCR 1.70	BCR 4.10
Banz et al. [51]	1-2 years	2	2-dose vaccination at 11–15 years^a^	BCR 0.30; EUR 856/LYG	BCR 1.29
Beutels et al. [37]	15 months	1	No vaccination	BCR 0.82; EUR 14,700/LYG	BCR 4.60
	12 years^a^	1	No vaccination	BCR 1.94	BCR 6.02
Bilcke et al. [30]	1 year (95 % coverage)	1	No vaccination	EUR 550–14,140/QALY	NA
	1 year (1st dose, 95 % coverage); 4 years (2nd dose, 90 % coverage)	2	No vaccination	EUR 5,240-31,942/QALY	NA
	1 year (1st dose, 95 % coverage); 11 years (2nd dose, 80 % coverage)	2	No vaccination	EUR 5,043-29,775/QALY	NA
	1 year (1st dose, 50 % coverage); 4 years (2nd dose, 50 % coverage)	2	No vaccination	EUR 3,345-23,240/QALY	NA
	All vaccination options (including and excluding additional HZ vaccination)	2	No vaccination	Net QALY loss for many time horizons^b^; EUR 36,256-135,961/LYG^b^	NA
Bonanni et al. [36]	12-18 months	2	No vaccination	BCR 0.67	BCR 3.47
	13 years	2	No vaccination	BCR 0.36	BCR 2.60
Brisson et al. [31]	12 months	1	No vaccination	BCR 0.61; EUR 38,142/LYG	BCR 5.24
	12 months	1	No vaccination	BCR 0.59^c^; EUR 40,193/LYG^c^	BCR 5.09^c^
	12 months	1	No vaccination	BCR 0.16^b^; EUR 101,296/LYG^b^	NA
	12 years^a^	1	No vaccination	BCR 0.73; EUR 15,863/LYG	BCR 4.44
Brisson et al. [32]	Infants	1	No vaccination	Net QALY loss^b^	Net QALY loss^b^
	11 years^a^	1	No vaccination	EUR 26,110/QALY^b^	Cost-saving^b^
Coudeville et al. [52]	<6 years	1	No vaccination	Net benefit EUR 326.8 million	NA
Coudeville et al. [53]	12-36 months	1	No vaccination	BCR 1.20 at high vaccination coverage	BCR 3.50 at high vaccination coverage
Coudeville et al. [54]	12-36 months	1	No vaccination	Cost-saving at high vaccination coverage (Germany 51 %; France 6.7 %); EUR 6.960/LYG at low vaccination coverage (France; cost-saving in Germany)	Cost-saving at high vaccination coverage (Germany 61 %; France 60 %)
DIez Domingo et al. [55]	15 months	1	No vaccination	BCR 0.54	BCR 1.61
Getsios et al. [56]	12 months	1	No vaccination	EUR 71,722/QALY; EUR 36/varicella case avoided	Cost-saving
Ginsberg & Somekh [57]	12 months	1	No vaccination	BCR 1.63	BCR 19.33
Hammerschmidt et al. [58]	11-23 months (including a catch-up of 2–17 year olds^a^)	2 (1 dose for catch-up)	1-dose vaccination at 12–15 years^a^	BCR 1.08	BCR 2.56
Huse et al. [59]	15 months	1	No vaccination	NA	Cost-saving (net benefit of EUR 86.28 per vaccinee)
Lenne et al. [60]	1-2 years	1	No vaccination	BCR 0.91; EUR 5,202/LYG	BCR 3.70
Lieu et al. [61]	<6 years	1	No vaccination	BCR 0,90; EUR 21,648/LYG; EUR 5.68/varicella case prevented	BCR 5.40
Preblud et al. [62]	15 months	1	No vaccination	BCR 0.30	BCR 6.90 (including home care costs)
Scuffham et al. [63]	15 months	1	No routine vaccination but low private coverage	BCR 0.67	BCR 2.79
Scuffham et al. [34]	12 months	1	No vaccination	EUR 49.11/varicella case avoided; EUR 16,439/hospitalisation avoided	NA
	12 years^a^	1	No vaccination	EUR 404.81/varicella case avoided; EUR 26,791/hospitalisation avoided	NA
Thiry et al. [64]	11 years^a^	1	No vaccination	BCR 0.54; EUR 26,988/LYG	BCR 2.17
van Hoek et al. [33]	1 year (first dose); 3 years (second dose)	2	No vaccination	41 % of the simulations below GBP 20,000/QALY (EUR 26,576/QALY)^b^; 50 % of the simulations below GBP 30,000/QALY (EUR 39,864/QALY)^b^	NA
	1 year (first dose); 3 years (second dose) + HZ vaccination of the elderly	2	No vaccination	50 % of the simulations below GBP 20,000/QALY (EUR 26,576/QALY)^b^; 70 % of the simulations below GBP 30,000/QALY (EUR 39,864/QALY)^b^	NA
Zhou et al. [29]	Children	1	No vaccination	BCR 1.00	BCR 4.37
	Children	2	No vaccination	BCR 0.61	BCR 2.73
	Children	2	1-dose vaccination	BCR 0.13	BCR 0.56; EUR 95,584/QALY

*BCR* benefit-cost ratio; *LYG* life-year gained; *QALY* quality-adjusted life-year; *NA* not applicable

^a^With a negative or uncertain history of varicella

^b^Including the impact on HZ

^c^Including the impact of breakthrough varicella

BCRs **for two dose-vaccination** targeting young children ranged between 0.13 and 1.08 when adopting a health care payer perspective and between 0.56 and 3.47 when taking a societal perspective. The study by Bilcke et al. [30] reported ICERs below EUR 35,000 per QALY for a two dose-vaccination scheme from a payer perspective when assuming no exogenous boosting. The authors of this study found that a two-dose vaccination regime would be less cost-effective than a single-dose childhood vaccination strategy. Beyond that, the study by Zhou et al. [29] showed that the choice of the comparator was very influential when evaluating two-dose varicella vaccination. In the study by Bilcke et al. [30] a two-dose vaccination strategy led to more cost-effective results at lower (50 %) than at higher vaccination coverage (round 90 %) due to the development of herd protection effects.

The **inclusion of the impact on HZ incidence** led to less cost-effective results. Brisson et al. [31] found an ICER of EUR 101,296 per LYG (health care payer perspective) when evaluating one-dose vaccination for toddlers in Canada. Adopting a similar approach for England and Wales, Brisson et al. [32] found a net QALY-loss. In another study from the UK [33], which focused on a 2-dose schedule, 50 % of the simulations exceeded a threshold of GBP 30,000 per QALY (EUR 39,864 per QALY). Bilcke et al. [30] concluded that a childhood vaccination programme is not expected to be cost-effective for several decades when assuming exogenous boosting.

BCRs of **vaccinating young adolescents** ranged between 0.36 and 1.94 from a health care payer perspective. ICERs were EUR 15.863 per LYG [31], EUR 26,110 per QALY [32] and EUR 26,791 per hospitalisation avoided [34]. Compared to cost-effectiveness estimates of routine childhood varicella vaccination, two studies showed that adolescent vaccination strategies might be less cost-effective than targeting toddlers [35, 36], while other studies found contrary results [31, 32, 34, 37]. The inclusion of indirect costs (societal perspective) improved the BCRs as it was found with the toddler vaccination strategies.

#### Herpes zoster vaccination

The results of models assessing the cost-effectiveness of HZ vaccination are shown in Table 6. One study [38] reported cost-effectiveness results for scenarios most and least in favour of vaccination instead of reporting results of a base-case analysis. Hence, the results of this study comprised a wide range of estimates ranging from EUR 1,200 to 291,240 per QALY (payer perspective). When considering a payer perspective, ICERs among all other studies ranged from EUR 5,412 to 140,125 per QALY. However, the majority of studies reported ICERs from EUR 10,000 to 40,000 per QALY. In terms of costs per HZ case avoided, ICERs varied from EUR 584 to 42,164 in the study from Bilcke et al. [38] and from EUR 817 to 9,433 in other studies when adopting a payer perspective. Few studies also reported ICERs in terms of costs per PHN-case avoided, which ranged from EUR 2,936 to 35,717 (payer perspective).

**Table 6:** Economic results of the models evaluating HZ vaccination in the elderly (2010 EUR, German price level)

Study	Age at vaccination (years)	Comparator	Health care payer perspective	Societal perspective
Annemans et al. [65]	50+	No vaccination	EUR 6,624/QALY; EUR 1,046/HZ case avoided; EUR 3,495-3,523/PHN case avoided^a^	EUR 6,822/QALY; EUR 1,077/HZ case avoided; EUR 3,600-3,629/PHN case avoided^a^
	60+	No vaccination	EUR 6,809/QALY; EUR 1,310/HZ case avoided; EUR 3,942-3,969/PHN case avoided^a^	EUR 7,148/QALY; EUR 1,375/HZ case avoided; EUR 4,039-4,137/PHN case avoided^a^
	65+	No vaccination	EUR 7,184/QALY; EUR 1,560/HZ case avoided; EUR 4,176-4,336/PHN case avoided^a^	EUR 7,577/QALY; EUR 1,645/HZ case avoided; EUR 4,404-4,574/PHN case avoided^a^
	60-64	No vaccination	EUR 5,694/QALY; EUR 817/HZ case avoided; EUR 2,936-2,969/PHN case avoided^a^	EUR 5,867/QALY; EUR 842/HZ case avoided; EUR 3,025-3,059/PHN case avoided^a^
	65-69	No vaccination	EUR 5,412/QALY; EUR 873/HZ case avoided; EUR 2,967-2,991/PHN case avoided^a^	EUR 5,628/QALY; EUR 909/HZ case avoided; EUR 3,087-3,112/PHN case avoided^a^
	60-69	No vaccination	EUR 5,553/QALY; EUR 844/HZ case avoided; EUR 2,951-2,980/PHN case avoided^a^	EUR 5,747/QALY; EUR 874/HZ case avoided; EUR 3,054-3,085/PHN case avoided^a^
Bilcke et al. [38]	60	No vaccination	EUR 1,200-46,968/QALY; EUR 584–5,148/HZ case avoided	NA
	70	No vaccination	EUR 2,200-70,496/QALY; EUR 1,239-8,603/HZ case avoided	NA
	80	No vaccination	EUR 3,824-126,793/QALY; EUR 2,867-17,353/HZ case avoided	NA
	85	No vaccination	EUR 5,272-291,240/QALY; EUR 4,451-42,164/HZ case avoided	NA
Bilcke et al. [30]	50 or 60	No vaccination	No results for a sole HZ vaccination reported; see Table 5 for results on combined varicella and HZ vaccination	NA
Bresse et al. [66]	65+	No vaccination	EUR 11,480/QALY; EUR 2,479/HZ case avoided; EUR 4,101/PHN case avoided^b^	NA
	70-79	No vaccination	EUR 8,876/QALY; EUR 2,090/HZ case avoided; EUR 3,302/PHN case avoided^b^	NA
Brisson et al. [67]	50	No vaccination	EUR 36,667/QALY	NA
	60	No vaccination	EUR 26,563/QALY	NA
	65	No vaccination	EUR 24,002/QALY	NA
	70	No vaccination	EUR 22,924/QALY	NA
	80	No vaccination	EUR 33,153/QALY	NA
de Boer et al. [40]	60	No vaccination	EUR 40,050/QALY	EUR 33,901/QALY
	65	No vaccination	EUR 34,440/QALY	EUR 33,511/QALY
	70	No vaccination	EUR 28,491/QALY	EUR 28,284/QALY
	75	No vaccination	EUR 28,506/QALY	EUR 28,506/QALY
Edmunds et al. [68]	65	No vaccination	EUR 5,435-100,700/QALY^c^	NA
Hornberger et al. [28]	69	No vaccination	NA	From cost-saving up to EUR 250,470/QALY^d^
Moore et al. [69]	50+	No vaccination	EUR 17,681/QALY; EUR 1,957/HZ case avoided; EUR 7,369-7,413/PHN case avoided	EUR 15,520/QALY; EUR 1,710/HZ case avoided; EUR 6,434-6,472/PHN case avoided
	50-54	No vaccination	EUR 18,041/QALY	EUR 12,488/QALY
	55-59	No vaccination	EUR 16,182/QALY	EUR 12,124/QALY
	60-64	No vaccination	EUR 14,931/QALY	EUR 12,866/QALY
	65-69	No vaccination	EUR 13,967/QALY	EUR 13,638/QALY
	70-74	No vaccination	EUR 17,814/QALY	EUR 17,814/QALY
	75-79	No vaccination	EUR 20,352/QALY	EUR 20,352/QALY
	80-84	No vaccination	EUR 27,176/QALY	EUR 27,176/QALY
	85-89	No vaccination	EUR 45,799/QALY	EUR 45,799/QALY
	90-94	No vaccination	EUR 67,522/QALY	EUR 67,522/QALY
	95-99	No vaccination	EUR 100,562/QALY	EUR 100,562/QALY
	100+	No vaccination	EUR 140,125/QALY	EUR 140,125/QALY
Najafzadeh et al. [26]	60+	No vaccination	EUR 28,314/QALY	NA
	60-74	No vaccination	EUR 24,002/QALY	NA
	75+	No vaccination	EUR 44,123/QALY	NA
Pellissier et al. [70]	60+, general population	No vaccination	EUR 16,170/QALY	EUR 14,232/QALY
	60+, immunocompetent only	No vaccination	EUR 24,211/QALY	EUR 22,255/QALY
Rothberg et al. [42]	60, male	No vaccination	NA	EUR 130,097/QALY
	60, female	No vaccination	NA	EUR 81,076/QALY
	70, male	No vaccination	NA	EUR 59,794/QALY
	70, female	No vaccination	NA	EUR 39,512/QALY
	80, male	No vaccination	NA	EUR 173,224/QALY
	80, female	No vaccination	NA	EUR 111,779/QALY
Szucs et al. [71]	70-79	No vaccination	EUR 13,743/QALY; EUR 3,565/HZ case avoided; EUR 8,334/PHN case avoided	CHF 15,361/QALY; CHF 3,985/HZ case avoided; EUR 9,315/PHN case avoided
Ultsch et al. [39]	50	No vaccination	EUR 37,173/QALY; EUR 1,587/HZ case avoided; EUR 32,545/PHN case avoided	EUR 30,901/QALY; EUR 1,320/HZ case avoided; EUR 27,054/PHN case avoided
	55	No vaccination	EUR 32,480/QALY; EUR 1,518/HZ case avoided; EUR 26,194/PHN case avoided	EUR 28,244/QALY; EUR 1,320/HZ case avoided; EUR 22,777/PHN case avoided
	60	No vaccination	EUR 30,212/QALY; EUR 1,525/HZ case avoided; EUR 22,337/PHN case avoided	EUR 28,146/QALY; EUR 1,419/HZ case avoided; EUR 20,809/PHN case avoided
	65	No vaccination	EUR 30,807/QALY; EUR 1,655/HZ case avoided; EUR 20,951/PHN case avoided	EUR 29,526/QALY; EUR 1,586/HZ case avoided; EUR 20,079/PHN case avoided
	70	No vaccination	EUR 42,190/QALY; EUR 2,732/HZ case avoided; EUR 22,813/PHN case avoided	EUR 41,942/QALY; EUR 2,716/HZ case avoided; EUR 22,679/PHN case avoided
	75	No vaccination	EUR 55,171/QALY; EUR 3,939/HZ case avoided; EUR 27,396/PHN case avoided	EUR 54,940/QALY; EUR 3,923/HZ case avoided; EUR 27,281/PHN case avoided
	80	No vaccination	EUR 92,734/QALY; EUR 9,433/HZ case avoided; EUR 35,717/PHN case avoided	EUR 92,541/QALY; EUR 9,414/HZ case avoided; EUR 35,643/PHN case avoided
van Hoek et al. [72]	60	No vaccination	EUR 36,302/QALY	NA
	65	No vaccination	EUR 27,747/QALY	NA
	70	No vaccination	EUR 20,589/QALY	NA
	75	No vaccination	EUR 25,211/QALY	NA
van Hoek et al. [33]	75	No vaccination	49 % of the simulations below GBP 20,000/QALY (EUR 26,576/QALY); 96 % of the simulations below GBP 30,000/QALY (EUR 39,864/QALY)	NA
	75 and 2-dose varicella vaccination of children	No vaccination	50 % of the simulations below GBP 20,000/QALY (EUR 26,576/QALY); 70 % of the simulations below GBP 30,000/QALY (EUR 39,864/QALY)	NA
van Lier et al. [41]	60	No vaccination	EUR 39,577/QALY	EUR 37,638/QALY
	65	No vaccination	EUR 30,514/QALY	EUR 30,514/QALY
	70	No vaccination	EUR 21,219/QALY	EUR 21,219/QALY
	75	No vaccination	EUR 23,779/QALY	EUR 23,779/QALY
	80	No vaccination	EUR 33,661/QALY	EUR 33,661/QALY

*HZ* herpes zoster; *PHN* post-herpetic neuralgia; *QALY* quality-adjusted life-year; *NA* not applicable

^a^Depending on the duration of PHN

^b^All results from the third-party payer perspective

^c^Depending on the efficacy and the duration of protection

^d^Depending on vaccination costs

When taking a societal perspective, one US study [28] reported a wide range of results ranging from cost-saving to EUR 250,470 per QALY. Results of other studies varied from EUR 5,628 to 173,224 per QALY.

Several studies, which included waning of vaccine-induced immunity and reported results for different ages at vaccination identified an U-shaped figure of vaccination age-related ICERs: Cost-effectiveness ratios decreased with increasing age at vaccination up to the age of 60 or 70 years and then increased with further increase in age at vaccination. In addition, many studies reported that cost-effectiveness was highly dependent on the assumed duration of vaccine-induced protection and the price of the vaccine. Particularly, the study by Hornberger et al. [28] showed how strongly ICERs can be affected by changes in duration of protection and vaccine cost. Ultsch et al. [39] also found a considerable impact when exploring the combined influence of varying waning immunity rates and durations of stable vaccine efficacy.

The majority of the included studies concluded that HZ vaccination would represent a cost-effective strategy. However, some studies came to different conclusions, mostly due to the application of different cost-effectiveness thresholds. The authors of a Dutch study [40] concluded that HZ vaccination might be cost-effective when using a threshold of EUR 50,000 per QALY, but not when decreasing the threshold to EUR 20,000 per QALY. Another study from the Netherlands [41] also reported ICERs above EUR 20,000 per QALY. The authors of this study concluded that HZ vaccination at the age of 70 years is expected to be marginally cost-effective. Hornberger et al. [28] were very cautious in deriving clear conclusions because of the high uncertainty around the cost-effectiveness results. The same applies to the US study by Rothberg et al. [42]. They found that ICERs often exceeded USD 100,000 (EUR 80,000-90,000) per QALY.

#### Combined varicella and herpes zoster vaccination strategy

Two studies [30, 33] modelled the cost-effectiveness of a combined varicella and HZ vaccination strategy (Table 5). The UK study [33] found that 70 % of the simulations lay below GBP 30,000 (EUR 39,864) per QALY when taking an infinite time horizon. Without the HZ vaccination component this fraction of simulations decreased to 50 %. According to the authors, the combined strategy is likely to be the optimum strategy, but results were highly sensitive with regard to the applied time frame. For example, when adopting time horizons of 30 to 50 years, there was a high probability that the combined strategy would not be cost-effective. The Belgium model adaptation [30], which was based on the previously mentioned UK model, predicted that a combined vaccination strategy would lead to a net QALY loss for many time horizons.

## Discussion

### Key findings

This systematic review was conducted to summarise the current state of evidence on the cost-effectiveness of varicella and HZ vaccination in high-income countries. To our knowledge, this is the first systematic review covering cost-effectiveness studies of both varicella and HZ vaccination. The major findings are outlined below:

#### Varicella vaccination

- When ignoring the potential impact on HZ and adopting a health care payer perspective, universal childhood varicella vaccination was usually a cost-effective or even cost-saving strategy.
- When switching to a societal perspective, childhood varicella vaccination was found to be a cost-saving intervention.
- Vaccination of adolescents was found to be a cost-effective or cost-saving strategy. However, it remains unclear if adolescent vaccination is more or less cost-effective than childhood vaccination due to inconsistent study results.
- Taking the potential impact on HZ into account, it is doubtful that childhood varicella vaccination appears to be cost-effective, at least for several decades.

#### HZ vaccination

- In most studies, HZ vaccination was predicted to be cost-effective or marginally cost-effective.
- When considering both a payer and a societal perspective, the differences in results between the two perspectives decreased with increasing age at vaccination since indirect costs due to sick leave become less relevant in the elderly population.
- When waning of vaccine-induced immunity was modelled, cost-effectiveness of HZ vaccination was highly dependent on the age at vaccination. ICERs decreased with increasing age at vaccination up to a certain age, followed by a re-increase of the ICERs (U-shape) for older ages. Most results suggest that the optimal age for HZ vaccination is between 60 and 70 years or around 70 years. Furthermore, cost-effectiveness was dependent on the price of the vaccine, the duration of protection and the assumed cost-effectiveness threshold.

### Choice of the model

Model choice can influence the results to a great extent when evaluating the cost-effectiveness of vaccines. In static models such as decision trees and Markov models the force of infection is constant over time because individuals were not allowed to interact with each other. In contrast, dynamic models account for interactions between individuals and therefore the force of infection depends on the number of susceptible, infectious and recovered individuals in the population. This is why dynamic models can include herd protection effects when evaluating the impact of vaccines.

More than half of the studies evaluating varicella vaccination were based on dynamic models and took herd protection effects into account. This is an important requirement to assess the impact of different coverage rates. In several studies the varicella vaccination uptake was found to be one of the most influential parameters. Since the force of infection for HZ is constant by nature, the models assessing only HZ vaccination were kept static.

### Exogenous boosting

The results of studies evaluating the cost-effectiveness of varicella vaccination were very sensitive to the structural assumption of allowing for exogenous boosting and its consequences on HZ incidence. Varicella vaccination was found to be a cost-effective or cost-saving strategy as long as the potential impact on HZ incidence was ignored. When taking the potential impact on HZ incidence into account, varicella vaccination was unlikely to be cost-effective. However, the study by van Hoek et al. [33] showed that the negative effect on HZ could, at least partly, be mitigated by the implementation of parallel (temporally limited) HZ vaccination of the elderly.

### Indirect costs

Many of the studies on varicella vaccination underlined the role of indirect costs for the assessment of cost-effectiveness. When adopting a societal perspective, savings were largely due to the inclusion of indirect costs. A previously published review of cost-effectiveness studies on varicella vaccination [43] found that the indirect costs ranged between 42 and 98 % of the total costs. On the contrary, results of models evaluating the cost-effectiveness of HZ vaccination were less sensitive to the cost perspective adopted because in many scenarios the target population belonged to age groups with a low level of labour market participation.

### Comparison with previous reviews

We are aware of four previously published systematic reviews on studies assessing the cost-effectiveness of varicella vaccination [43–46] and one review on studies examining the cost-effectiveness of HZ vaccination exclusively [47].

Thiry et al. [44] concluded that universal vaccination of healthy children would generate cost savings to society. Although some of the included studies are based on dynamic models which could account for herd immunity effects, only one of the (subsequently added) studies covered the potential impact on HZ. The results of this study were only in accordance with the findings of the other studies when ignoring the impact on HZ. Incorporating the impact on HZ led to highly inefficient results. Rozenbaum et al. [45] found that routine childhood vaccination was a cost-effective or even a cost-saving strategy as long as the potential impact on HZ was not considered in the model analyses. Cost-savings were generally driven by the inclusion of indirect costs in terms of production losses. Rozenbaum et al. [45] attached great importance to point out that the benefits provided by the implementation of early-childhood varicella vaccination might be offset due to an increase in HZ cases in the elderly population. Therefore, they suggested varicella vaccination of high-risk groups such as susceptible adolescents only, as long as the interactions between varicella and HZ are not clarified. The conclusions of the review by de Soárez et al. [43] were consistent with those of the two previously published reviews [44, 45]. Interestingly, Unim et al. [46] concluded that the results of the reviewed studies undoubtedly support the introduction of a universal varicella vaccination programme. Although the authors included two studies accounting for the potential impact on HZ [31, 32], the implications of such negative effects of a childhood varicella vaccination programme were not discussed by Unim et al. [46]. Hence, the conclusions of this review are highly disputable. Szucs et al. [47] found that in almost all studies HZ vaccination was considered as a cost-effective intervention. Amongst others, age at vaccination and vaccination costs had a great impact on the results. This conclusion is in line with findings of our review.

One of the strengths of our systematic review is that it is more comprehensive than previously published review articles. We included economic evaluations of varicella vaccination and HZ vaccination while other reviews have been focused on one of the two vaccines at a time. A combined assessment of the cost-effectiveness of varicella and HZ vaccination programmes gives consideration to the close relationship of both diseases.

### Limitations

Our review was focused on models evaluating varicella vaccination in children and adolescents as well as HZ vaccination in the elderly. Studies limited to varicella vaccination of specific target groups such as health care workers or pregnant women were excluded because the current debate is more about whether universal varicella and HZ vaccination is good value for money; still the consideration of specific risk groups can affect the overall cost-effectiveness. Furthermore, we excluded studies which provided no sufficient description of the used methods (e.g. Hudeckova et al. [48]; Gialoretti et al. [49]); such studies might provide valid results but could not be evaluated. In addition, only one literature database (PubMed) was searched, and our review was restricted to articles written in English or German and to studies reporting results for high-income economies.

It is well known that transferability of results of cost-effectiveness analyses across countries is usually affected by a wide range of factors such as variation in disease epidemiology, clinical practice patterns, unit costs, other health care characteristics or methodological decisions [50]. This limitation also holds true for the reviewed studies. For example, there is a large variability in vaccination costs between the included studies. Nevertheless, many of the studies reported similar results and drew consistent conclusions.

## Conclusions

Cost-effectiveness of childhood varicella vaccination rests to a large extent on the interaction between varicella and HZ. When assuming no exogenous boosting of HZ immunity, varicella vaccination can be considered as a cost-effective or a cost-saving strategy. However, this conclusion needs to be revised when assuming that exogenous boosting exists because the inclusion of the effects of exogenous boosting leads to less favourable results. In this situation, the overall cost-effectiveness seems to become more favourable when routine childhood varicella vaccination is accompanied by the (temporary) implementation of HZ vaccination in the elderly. As a consequence, clarification on the role of exogenous boosting is crucial for decision-making regarding varicella vaccine introduction. Cost-effectiveness of HZ vaccination itself is mainly dependent on the chosen age at vaccination, the price of the vaccine and the magnitude of the cost per QALY threshold.

Based on this review we identified several important issues that need to be considered when evaluating the health economic impact of varicella and/or HZ vaccination. Future economic evaluations of varicella vaccination should apply a dynamic modelling approach because only dynamic models can take into account herd protection effects and the potential impact of varicella vaccination on HZ incidence due to reduced or absent exogenous boosting. In contrast, when the analysis is focused exclusively on HZ vaccination, a static model seems to be adequate since no change in the force of infection in HZ will be derived from the introduction of HZ vaccination. In addition, since fatality rates of varicella and HZ are rather low, we believe using life years gained (LYG) as an outcome parameter is not sufficient. Thus, we recommend, as done in most existing studies, to consider health-related quality of life outcome parameters (e.g. QALYs). Moreover, waning of vaccine-induced immunity seems to play an important role for the impact of both vaccines. Hence, we recommend addressing this issue already in the base-case analysis of future evaluations (instead of including waning in sensitivity analysis only). Furthermore, when considering waning of vaccine-induced immunity, the effects of administering a booster vaccine should be captured in sensitivity analysis because a booster shot might have significant impact on the results.
